# Study on the application and imaging characteristics of optical coherence tomography in vulva lesions

**DOI:** 10.1038/s41598-022-07634-1

**Published:** 2022-03-07

**Authors:** Lida Xu, Qian Ma, Shaochong Lin, Juan Ju, Shuo Feng, Zhongna Shi, Yang Bai, Junzhai Song, Junpeng Du, Baojin Wang

**Affiliations:** 1 Department of Gynecology, The Third Affiliated Hospital of Zhengzhou University, Henan International Joint Laboratory of Ovarian Malignancies, Zhengzhou, 450052 China; 2 Department of Pediatric Surgery, The Third Affiliated Hospital of Zhengzhou University, Zhengzhou, 450052 China

**Keywords:** Adaptive clinical trial, Translational research

## Abstract

In this study, a prospective study was conducted by using optical coherence tomography (OCT) in the in vivo detection of vulvar diseases. The clinical efficacy of the OCT we investigated in the detection of vulvar diseases, and the characteristics of the OCT images were defined. Overall, this study recruited 63 patients undergoing the colposcopy for vulvar lesions in three Chinese hospitals from December 20th, 2018 and September 24th, 2019. The colposcopy and the OCT examination were performed successively, and the OCT images were compared with the relevant tissue sections to characterize different lesions. The OCT diagnoses where categorized into 7 types, including normal and inflammatory vulva, condyloma acuminata, papilloma, lichen sclerosus, atrophic sclerosing lichen, fibrous epithelial polyp as well as cysts. The structural characteristics of the vulva tissue can be clearly observed in the OCT image, which are consistent with the characteristics of the tissue section. Compared with the pathological results, the sensitivity, specificity and accuracy of the OCT examination reached 83.82% (95% confidence interval, CI 72.5%–91.3%), 57.89% (95% CI 34.0%–78.9%) and 78.16%, respectively. The OCT is found with the advantages of being noninvasive, real-time and sensitive and with high resolution. It is of high significance to screening vulva diseases, and it is expected as one of the methods to clinically diagnose vulva diseases.

## Introduction

Vulva diseases consist of hypopigmentation disease, benign tumor and malignant tumor. Vulva hypopigmentation refers to a group of common and refractory skin diseases with pruritus as the main symptom, which is primarily characterized by hypopigmentation of vulva skin. It largely covers Lichen simplex chronicus (LSC), Lichen sclerosus (LS), Lichen planus (LP) and other vulva hypopigmentation diseases. As indicated from the epidemiological investigation, the disease has 1‰ ~ 3‰ incidence, and the malignant change rate ranges from 2% ~ 5%, which increases with age. LS can advance to facultative precancerous lesions of squamous cell carcinoma. Females with sclerosing lichen are subject to a 2% to 5% risk of vulva cancer. The biopsy can provide women with accurate information regarding the risk of vulva cancer^1,2^. Moreover, the biopsy helps distinguish sclerosing lichen planus from lichen planus and rule out the malignant transformation to squamous cell carcinoma^3^. The existing pathogenesis of this disease remains unclear, and effective radical cures have been rare. In general, the treatment methods comprise the conservative treatment, with the major aim to mitigate symptoms and prevent adhesions, as well as to manage long-term diseases, whereas the methods above have poor treatment effect and very high recurrence rates^4^.

Vulvar tumors include benign tumors, squamous intraepithelial lesions and malignant tumors. Over the past few decades, the overall incidence of vulvar cancer has been elevated by 4.6% every 5 years on average^5^. Squamous cell carcinoma is the most common malignancy of vulvae, in which intraepithelial neoplasia of vulvae is an important precursor, followed by vulva Paget's disease^6^.

The clinical diagnosis of vulvar lesions is largely determined by clinical manifestations. The most used method to diagnose vulvar lesions in clinical practice has been the routine gynecological examination, which is only employed for preliminary screening, whereas the nature of lesions cannot be accurately determined by naked eye observation only^3^. Though morphological and clinical examinations are key aspects of the diagnostic work, histological confirmation is applied as the gold standard for the diagnosis of any suspicious lesions. It refers to the only diagnostic method capable of diagnosing vulvar lesions at present^7^.

The electronic colposcopy refer to a procedure used to observe the skin changes and vascular morphology of the lesion site using intense light and magnification^8^. It can act as a non-invasive method to examine vulva lesions. Through the electronic colposcopy, small lesions difficult to observe by the naked eye can be identified. The addition of the aceto-whitening test helps identify vulva preccancerous lesions. Biopsies were performed under the colposcopy positioning to increase the detection rate. The effectiveness of the colposcopy combined with the acetic acid test in screening cervical precancerous lesions has been verified extensively. Vaginal intraepithelial neoplasia (VaIN) and vulvar intraepithelial neoplasia (VIN) have been increasingly diagnosed, which are often HPV-related and known precursors of cancer. The colposcopy of the vagina and vulva takes up an important part of screening for diseases of the lower reproductive tract^9^. The discovery of suspicious vulvar intraepithelial neoplasia (VIN)/ invasive lesions and ambiguous or refractory vulvar skin changes have been reported as the indications for 4–6 mm needle biopsy^10^.However, several studies proposed that challenges remain in colposcopy assessment of vulvar diseases^9^.

The OCT is a 3D imaging technology developed in the 1990s^11^. In Patel's study, OCT as a technology capable of high resolution imaging, with almost 10 times better depth resolution than high frequency ultrasound^12^. In addition, it can reconstruct the 3D image of internal structures of biological tissues through scanning. It has high resolution in transverse and axial dimensions, with the penetration depths of several millimeters^13^ and the imaging results comparable to histopathology. Since the imaging depth is identical to the scale covered by the conventional biopsy, the OCT may serve as an optical biopsy to guide interventional procedures, thereby reducing sampling errors and even replacing surgical biopsies^14^.

OCT is recognized as a novel imaging method for disease screening and diagnosis. OCT for real-time, in vivo and high-resolution tissue imaging and diagnosis has been extensively applied for diseases (e.g., ophthalmology^15^, skin^16^, cardiovascular^17^, and cancer^18^). Over the past few years, as OCT technology is advancing, its significance for the diagnosis and treatment of cervical lesions has aroused increasing attention. However, clinical data have been rare to support its application in the diagnosis and screening of vulvar lesions.

OCT is characterized by its high-resolution, real-time in vivo, high-speed noninvasive imaging that is capable of clearly presenting the internal structures of tissues. Gallwas et al.^14^ found that the sensitivity and specificity of the OCT in the diagnosis of cervical epithelial lesions ranged from 85 to 98%, and from 39 to 81%, respectively, whereas considerable false positive rates resulted in a decrease in the specificity of OCT. Zeng^19^ studied 497 3-D ultra-high-resolution optical coherence microscope (OCM) images and the relevant tissue HE staining sections from 159 isolated cervical specimens of 92 patients. These researchers could clearly distinguish single cells, normal cervix, ectropion, low-grade lesions, high-grade lesions, as well as infiltrating cancer. Furthermore, the sensitivity was 80% and the specificity was 89%.

In a multi-center clinical study conducted by Ren et al.^20^, the OCT images of cervical tissues in vivo were compared with the relevant histological images to summarize the optical characteristics of various cervical lesions in vivo and show satisfactory diagnostic results. In this study, a multicenter study as well, the OCT images of the vulva were obtained and compared with the related tissue images to summarize the optical characteristics of different vulva diseases. Subsequently, the results of the OCT were analyzed to discuss the application significance of the OCT for the diagnosis of vulvar diseases.

## Methods

### OCT system

**Figure 1:**
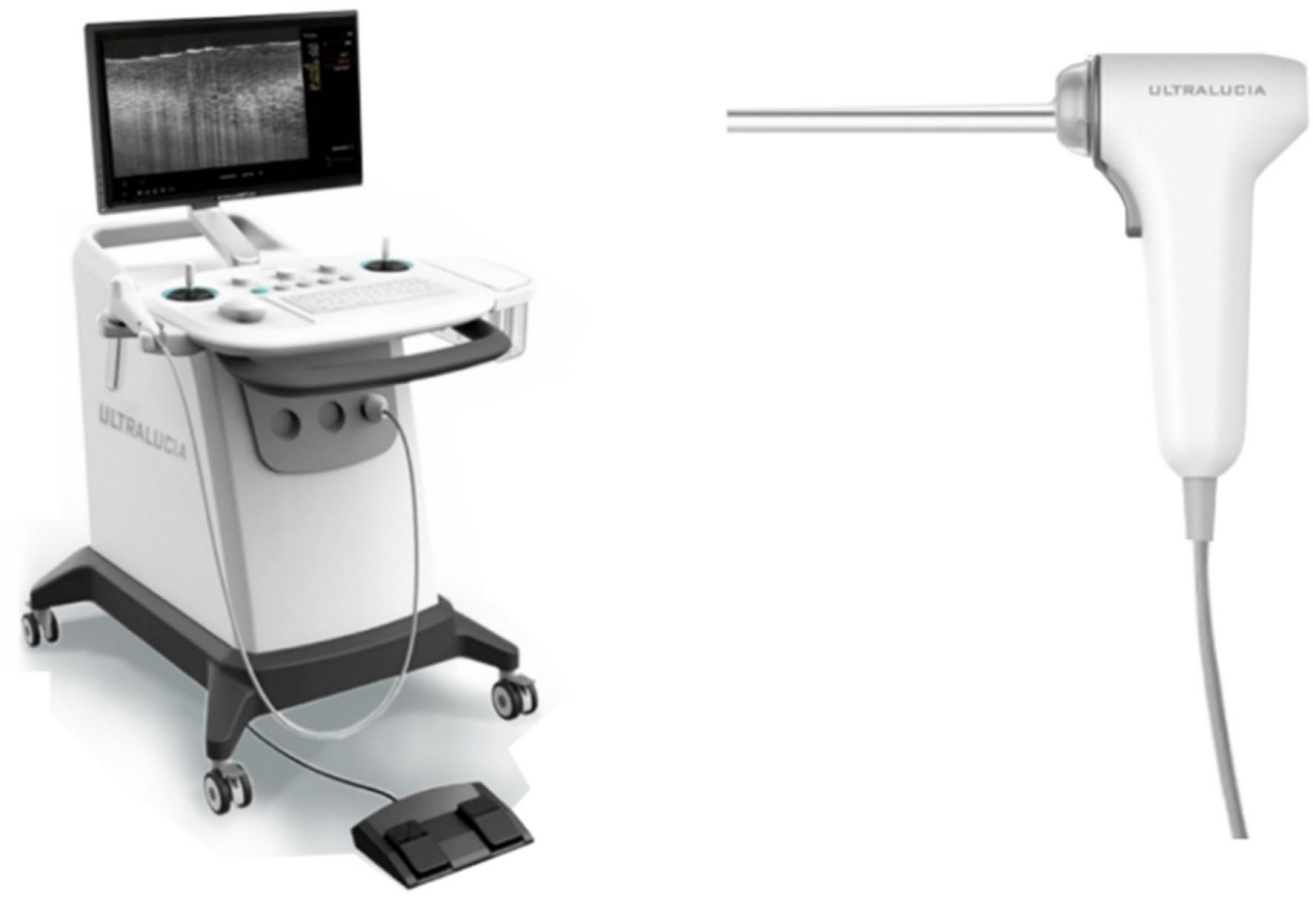
Ultralucia OCT cervical scanning system and handheld probe.

OCT cervical detector (MODEL: UL-C100) produced by Zhengzhou Opto-Ultrasonics Medical Technology Co., LTD was used for OCT examination in this study. A hand-held probe (Fig. 1) was adopted to deliver the imaging beam to the surface of the vulva lesion. To determine a wider scanning range, the image acquisition scanning mode was set to a circle. In this study, a new circular scanning mode was adopted for image acquisition, and the scanning diameter can be adjusted continuously from 0 to 2 mm according to the setting. Compared with the traditional linear scan mode, the circular scan mode could avoid the image distortion caused by sudden acceleration or deceleration of hardware. Most importantly, under the same hardware conditions, it could obtain a wider scan range and display more tissue information on the respective frame of OCT images. The OCT system is equipped with a wideband light source with a central wavelength of 850 nm. At an image speed of 20,000A/s, a circular scan with a diameter of nearly 1.18MM was conducted to capture an OCT image with an axial resolution of about 4 μm and a transverse resolution of 3–4 μm. The maximum scanning speed is 80,000 A scans/SEC, and the imaging depth is about 1 mm.

This study was approved by the Ethics Committee of the Third Affiliated Hospital of Zhengzhou University, the Ethics Committee of the First Affiliated Hospital of Xinxiang Medical College, and the Ethics Committee of Luohe Central Hospital. All studies were conducted complying with relevant guidelines/regulations. Informed consent was obtained from all participants and/or their legal guardians.

Sixty-three patients undergoing colposcopy examination for vulvar lesions in the gynecological outpatient department of the above three hospitals during December 20, 2018 and September 24, 2019 were recruited, and all of which had signed the informed consent. These hospitals include one provincial hospital and two municipal hospitals. All recruited patients had vulvar lesions and undergone the vulvar OCT examination and the vaginal biopsy by using the colposcopy. Table 1 lists the demographic information for the patients studied.

**Table 1:** Demographic information.

Hospital	No. of patients	Age (mean ± SD)	Colposcopy result
The Third Affiliated Hospital of Zhengzhou University	20	45.40 ± 14.41	Positive:17 Negative:3
The First Affiliated Hospital of Xinxiang Medical College	24	39.80 ± 11.77	Positive:24 Negative:0
Luohe Central Hospital	19	49.05 ± 11.16	Positive:19 Negative:0
Overall	63	44.37 ± 12.90	Positive:60 Negative:3

After signing the Agreement, the inspection process is presented below: Colposcopy: the bladder lithotomy position was taken to observe the surface structure and color of the vulva. For the lesions observed by using the colposcopy, the corresponding area was drawn out by solid line in the schematic diagram of the vulva. Dotted lines were used to circle the area where the subject feels abnormal, whereas no abnormality was observed by the colposcopy. The OCT examination: a disposable latex cover was installed on the handheld probe of the OCT system, which had no visual impact on the imaging. The disposable latex cover was supplied with the machine. The thickness was 0.6 mm in the unstretched state and 30 μm after stretching. Gently contact the surface of the vulva with the probe, so the key parts of the vulva tissue fell within the working distance of the probe. At least one OCT scanning point was selected for the respective anomaly area, and at least one OCT scanning point was taken for the area around each anomaly area. The scan sites were sequentially marked and OCT scans were performed point by point. After the OCT examination, the suspicious lesions found by using the colposcopy were taken from the relatively serious sites and sent for pathological examination.

### Image processing and comparison

OCT images were analyzed by using the Image processing software Image J. The OCT images were recalibrated to achieve a horizontal and axial aspect ratio of 1:1 for comparison with the corresponding histological sections. The H&E staining was scanned with a MoticEasyScan scanner to capture the digital images of the pathological sections. Among them, the MoticEasyScan scanner used is produced by China Xiamen McAudi Medical Diagnostic System Co., LTD. Moreover, OCT images captured from the corresponding biopsy site scans were processed. OCT images and corresponding histological images were placed side by side in PowerPoint in the same proportion. By carefully comparing the images, a well-structured region was taken from the histological image, and a region with the same area and similar tissue characteristics as the selected histological image was cut out from the corresponding OCT image. Thus, many well-matched OCT-H&E images were obtained. The process above was done by the data collector, independent of the three observers. However, since the biopsy site and the OCT scan site were not identical, the OCT image and the corresponding pathological tissue image might not originate from exactly the identical site. They usually shared common pathological characteristics for their close locations.

### OCT image classification and statistical analysis

Since there is no definite diagnostic feature of the OCT scan images of human vulva tissue, the statistical analysis on the corresponding OCT image features was conducted based on the classification of pathological results. Based on histopathological images, all histological and OCT images were classified into the 7 diseases (Table 2). Normal, inflammatory vulva was defined as negative, and pathological results complied with condyloma acuminata, papilloma, lichen sclerosus, lichen sclerosis et atrophicus, fibrous epithelial polyp as well as cyst were defined as positive. When the OCT image diagnosis of each biopsy site was consistent with the corresponding histological diagnosis and is positive, it would be defined as true positive. True negative was defined when OCT images and corresponding histological diagnosis were negative. The OCT images were viewed using Image J image processing software.

**Table 2:** OCT optical characteristics of different diseases.

CT result	Disease categories	No. of patients	Optical characteristics
Negative	Normal/in-flammation	28	The epithelium and stroma were well stratified, with clearly observed serrated rete pegs. The whole epithelium had a uniform scattering, and it was highly scattered. Striped glandular structures were seen in the underlying stroma. The pathological results showed that the tissue was inflammatory, and the morphology of the epithelial angle varied slightly due to the inflammatory stimulation. Considerable inflammatory cells infiltrated, resulting in the low-scattering stroma
Positive	Condyloma acuminata	2	The papillary projections of the epithelium could be clearly seen, and the overlying surface layer was hyperkeratinized, whereas the koilocyte was indistinguishable (pathological findings were visible)
Positive	Papilloma	4	The characteristic papillary projections could be clearly seen
Positive	Lichen sclerosus	16	The epithelium and stroma were well stratified, the overall scattering was uniform, the rete pegs disappeared, and no serrated structure was observed. The surface highlights were the keratinized layers
Positive	Atrophic sclerosing lichen	9	The epithelium and stroma were well stratified, the rete pegs disappeared, and the base flattened. However, compared with normal epithelium, the thickness of the epithelium was significantly thinner due to atrophy. Surface keratinization
Positive	Fibrous epithelial polyp	2	The dark strip area in the stroma increased obviously, which was suspected of capillaries. The rete peg is passivated and flattened
Positive	cyst	2	It presented a well-defined low-scattering dark area. The epithelium and stroma were stratified, and the scattering was uniform. As impacted by the compression of cysts, the rete pegs disappeared, and they were flattened. The surface layer keratinization appeared as a bright region with high scattering

As inspired by the study of Zeng^19^, a three-step training and blind test program was designed to assess the accuracy of diagnosis based on OCT scan images. Three investigators from three different institutes (investigators 1, an experienced OCT investigator, and investigators 2 and 3, experienced pathologists) participated in the training and testing. First, each investigator was provided with a dataset consisting of 50 OCT scan images and corresponding H&E staining images of pathological tissue sections. Through this data set, three investigators are trained. After they became familiar with and mastered the characteristics of OCT scan images, each investigator was provided with a pretest data set containing 50 individual OCT scan images. Each investigator was asked to diagnose OCT scan images based on the summarized image features. After the pre-test, each investigator's misclassified sample was informed of the corresponding correct diagnosis, and these data sets were thoroughly reviewed before the final blind test. In the final test, each investigator was provided with a dataset of OCT scan images collected in this study. Three investigators independently diagnosed each OCT image, and the third investigator examined the results. If the first two investigators gave different diagnoses, the third investigator would make the final diagnosis. H&E biopsies of biopsy samples were prepared and reviewed by local pathologists at each hospital. The OCT was compared with pathological results to analyze the sensitivity, specificity, and accuracy of the OCT^21^.The three investigators were unaware of the corresponding H&E biopsy and histopathological diagnosis during the pretest and final test. The sample size ratio for each category used in all three datasets is roughly the same, and these categories are included in the classifications in Table 2.

## Results

### OCT image features

OCT images clearly present the rete pegs of normal vulva, the legible layered structure of epithelium and stroma, and the structural changes of cysts, keratinized layers and rete pegs in the pathological vulva tissue, which were effectively consistent with the pathological examination results of the corresponding histological sections. As impacted by a disposable protective cover on the outside of the hand-held probe when the vulva tissue was scanned, the appearance of the protective cover and the outer surface of the probe on the top of the vulva tissue could be observed in the OCT image.

**Figure 2:**
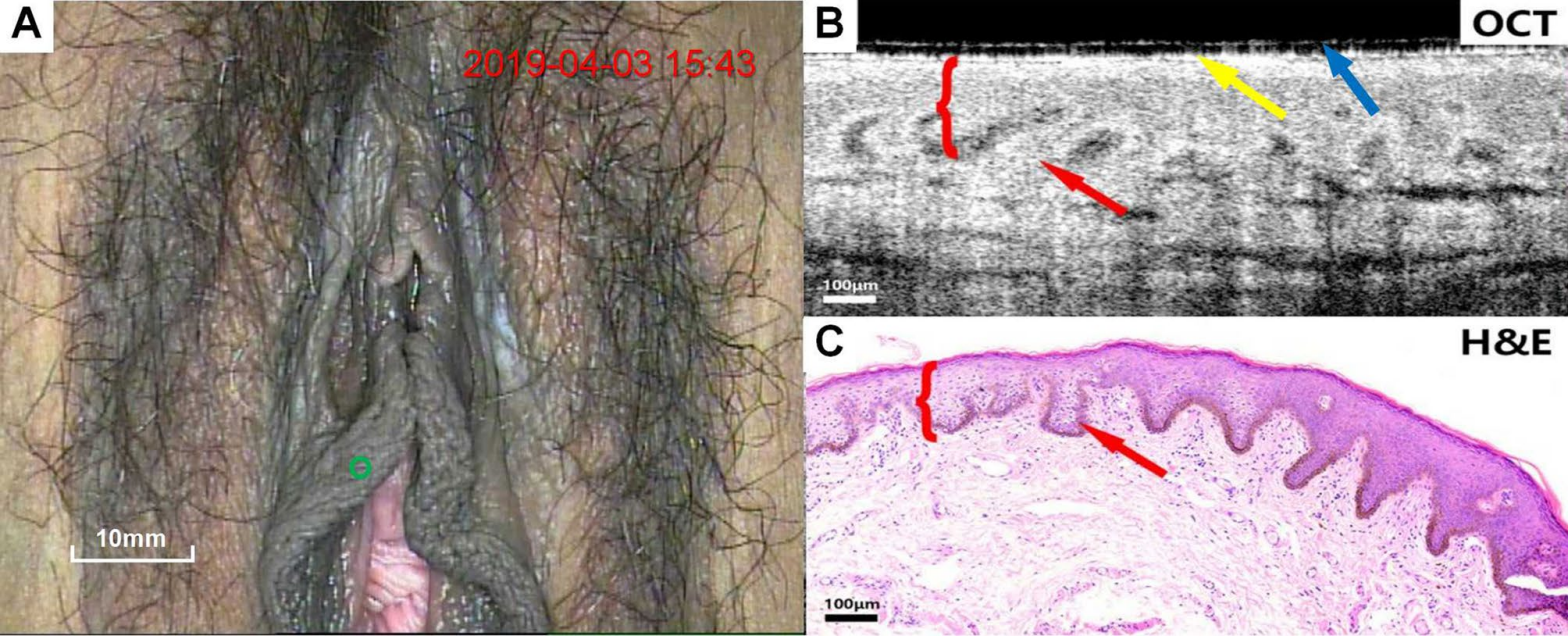
Typical normal vulvar colposcopy images, OCT images, and corresponding H&E staining images of vulvar tissue. Scale bars 100 μm.

Figure 2A–C present colposcopy image of normal vulva tissue, OCT images of normal vulva tissue and the corresponding H&E tissue sections. In normal vulva tissue, the green circles indicate the biopsy site and the OCT examination site (Fig. 2A) and the yellow arrow represents the outer surface of the probe (Fig. 2B). The blue arrow indicates the disposable latex protective layer in the OCT image (Fig. 2B). The epithelium and stroma show well-organized stratification on the OCT image, with clearly observed serrated rete pegs. The whole epithelium had a uniform scattering, and it was highly scattered. The striped glandular structures were identified in the underlying stroma.

**Figure 3:**
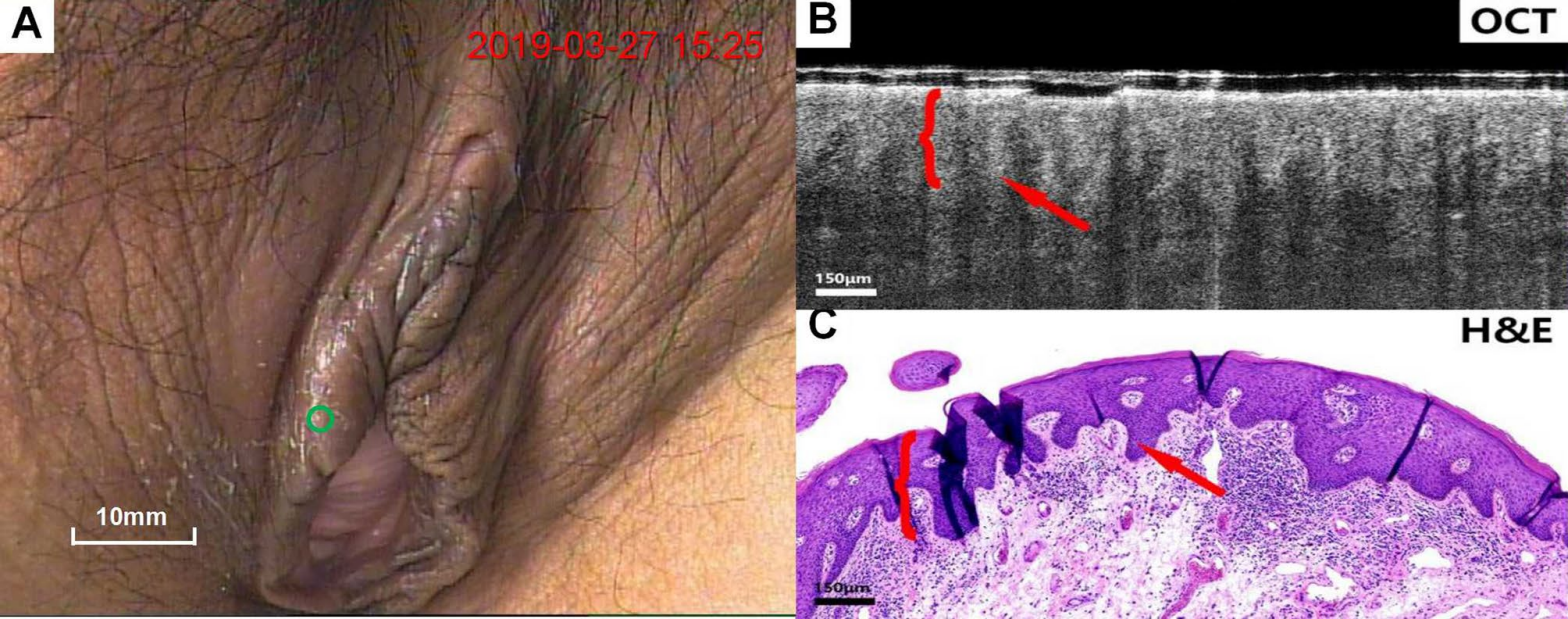
Colposcopy images, OCT images and H&E staining images of inflammatory vulva tissue. Scale bars 150 μm. OCT images of normal vulva (Fig. 2A–C) and inflammation (**A**–**C**), as well as the relevant pathological biopsy images, clearly demonstrate the epithelial-stromal boundary with serrated rete pegs.

Figure 3A–C present colposcopy images, OCT images and H&E staining images of inflammatory vulva tissue. The OCT images (Fig. 3B) showed marked epithelium and stroma stratification, and structures at rete pegs (arrows) could also be observed. The morphology of the rete pegs changes slightly due to inflammatory stimulation. Considerable inflammatory cells infiltrate, resulting in the low-scattering stroma.

**Figure 4:**
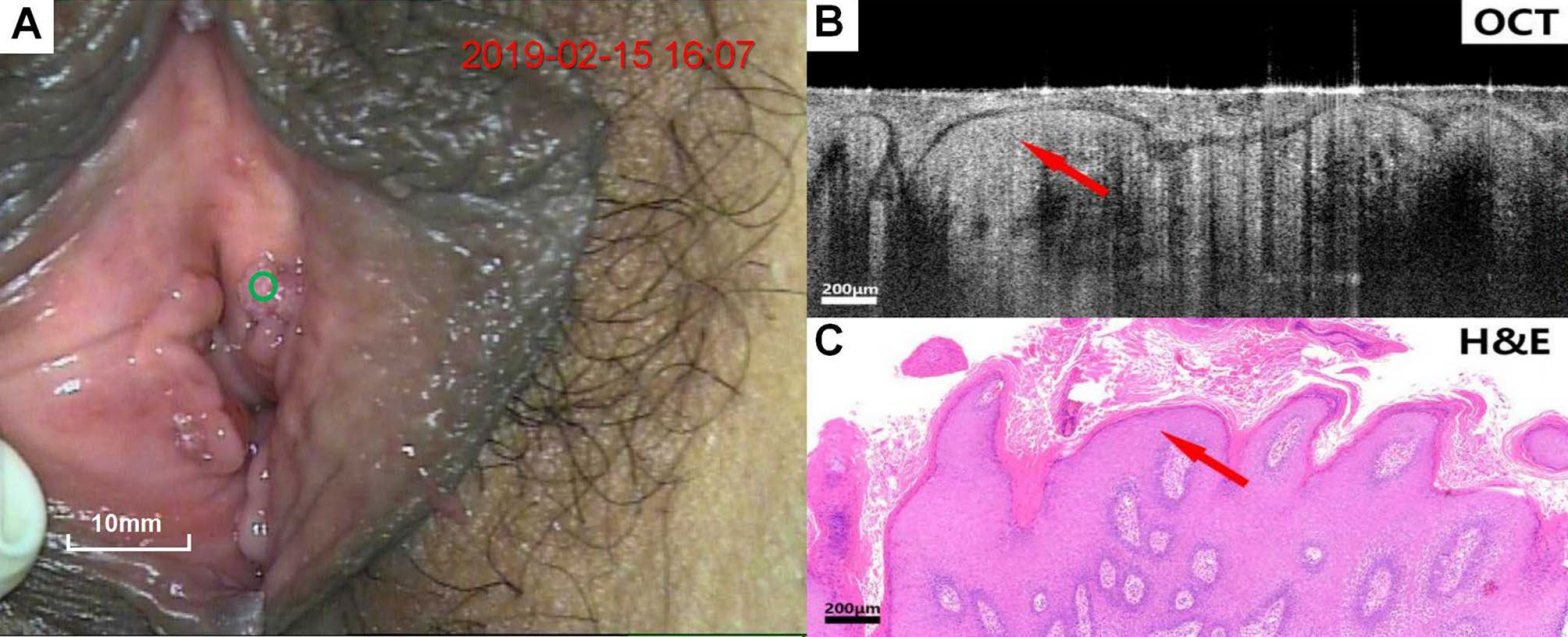
Colposcopy images, OCT images and H&E staining images of condyloma acuminata. Scale bars 200 μm.

**Figure 5:**
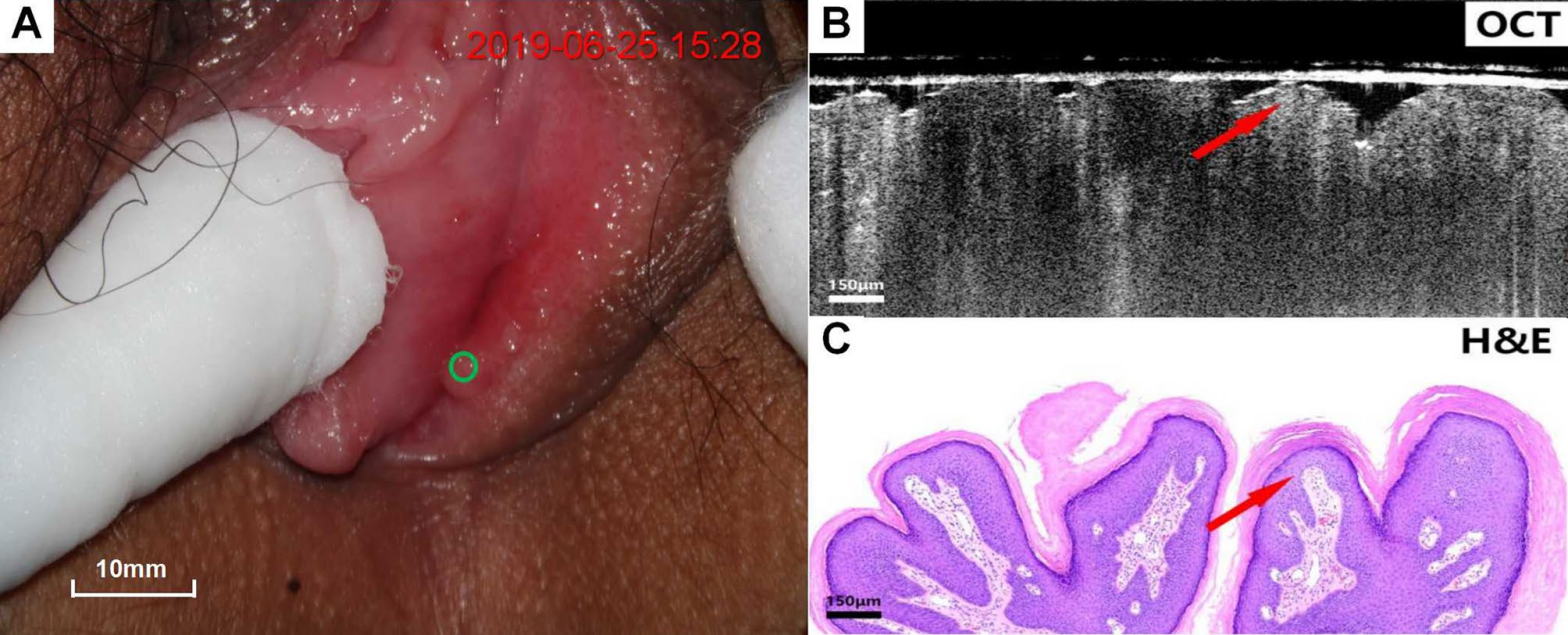
Colposcopy images, OCT images and H&E staining images of papilloma. Scale bars 150 μm. OCT images of condyloma acuminatum (Fig. 4B,C) and papilloma (**B**,**C**), as well as the corresponding pathological biopsy images, exhibit common features of papillary projections.

**Figure 6:**
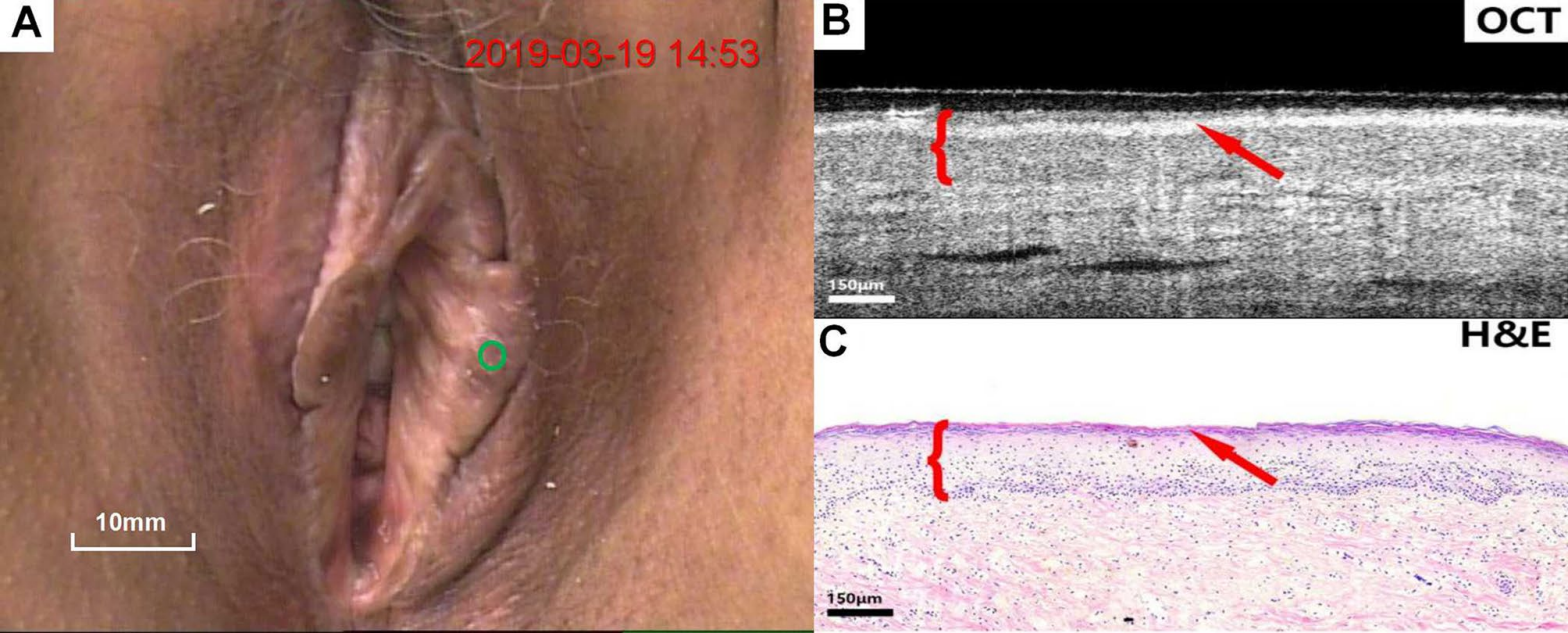
Colposcopy images, OCT images and H&E staining images of lichen sclerosus. Scale bars 150 μm.

**Figure 7:**
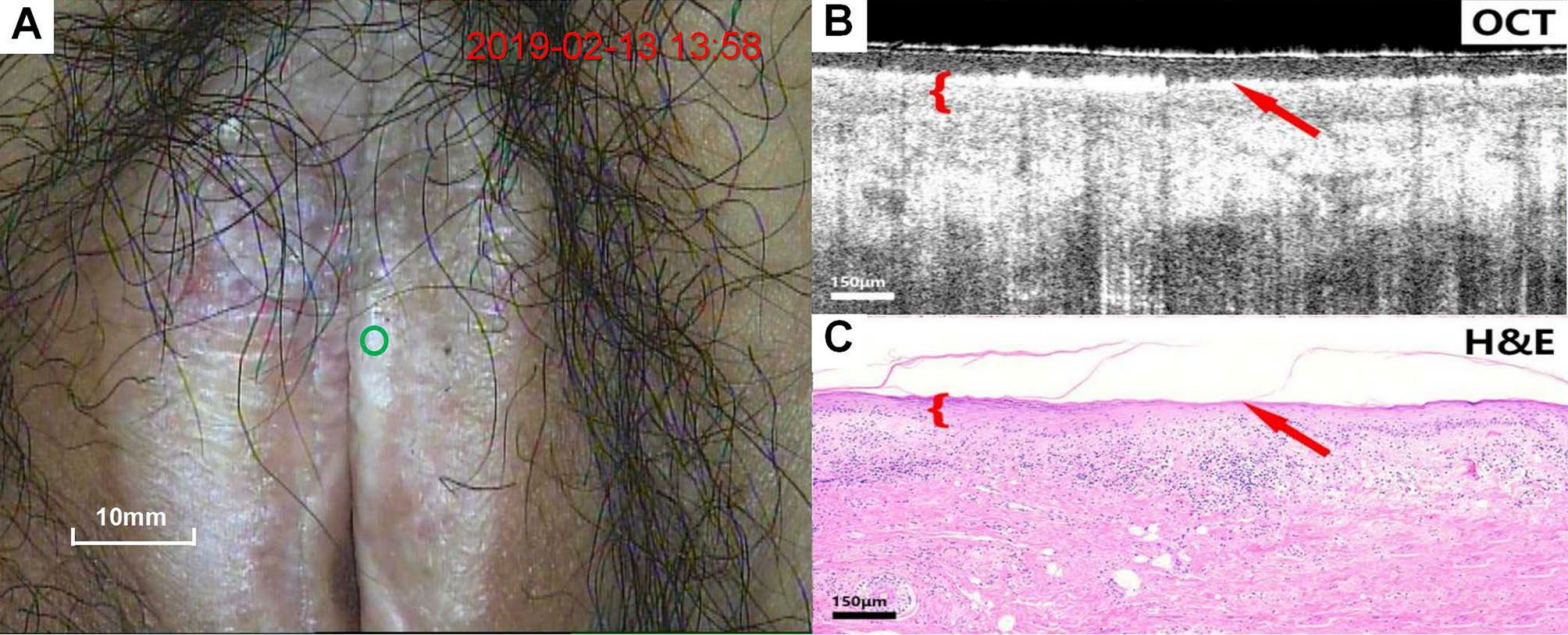
Colposcopy images, OCT images and H&E staining images of atrophic sclerosing lichen. Scale bars 150 μm. OCT images of sclerosing bryophytes (Fig. 6B,C) and atrophic sclerosing bryophytes (**B**,**C**), as well as the relevant pathological biopsy images, showed a uniform overall scattering and a good stratification. The rete peg was missing due to lichenosis. The atrophied epithelium was readily discernible on OCT images.

**Figure 8:**
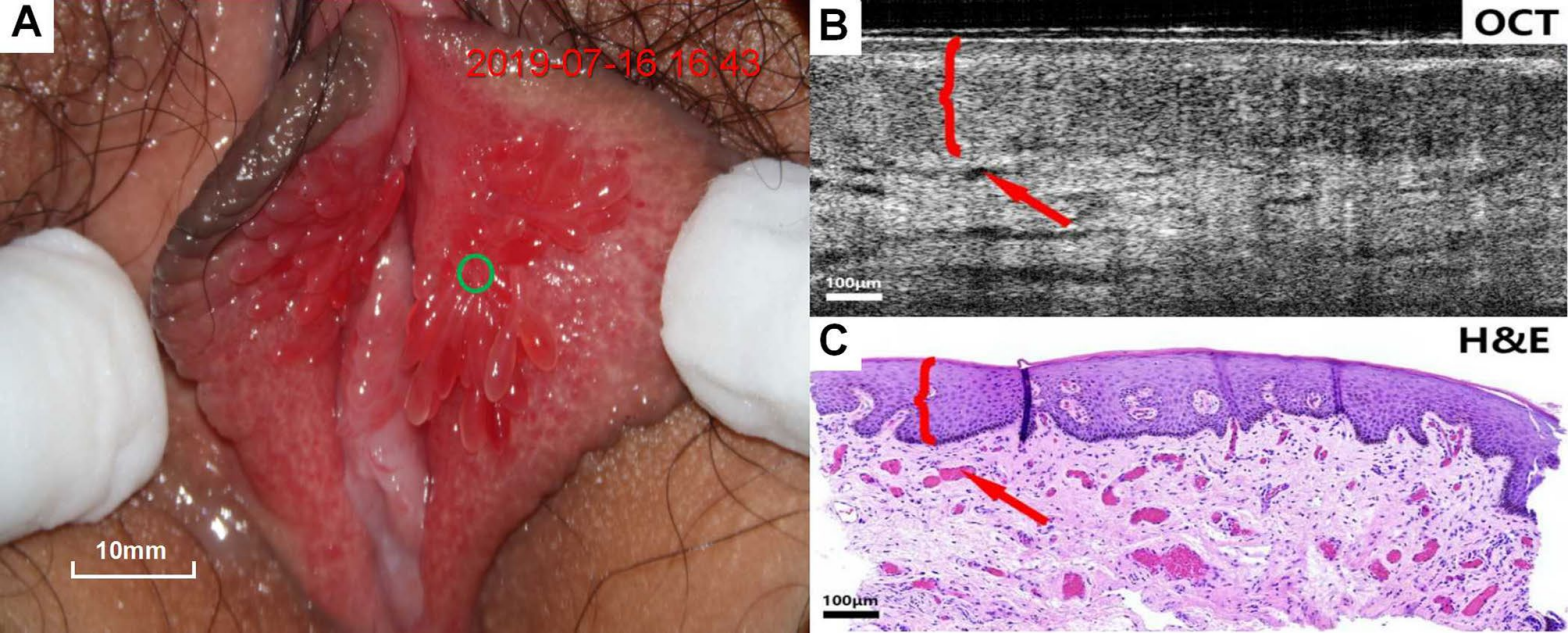
Colposcopy images, OCT images and H&E staining images of fibrous epithelial polyp. Scale bars 100 μm.

**Figure 9:**
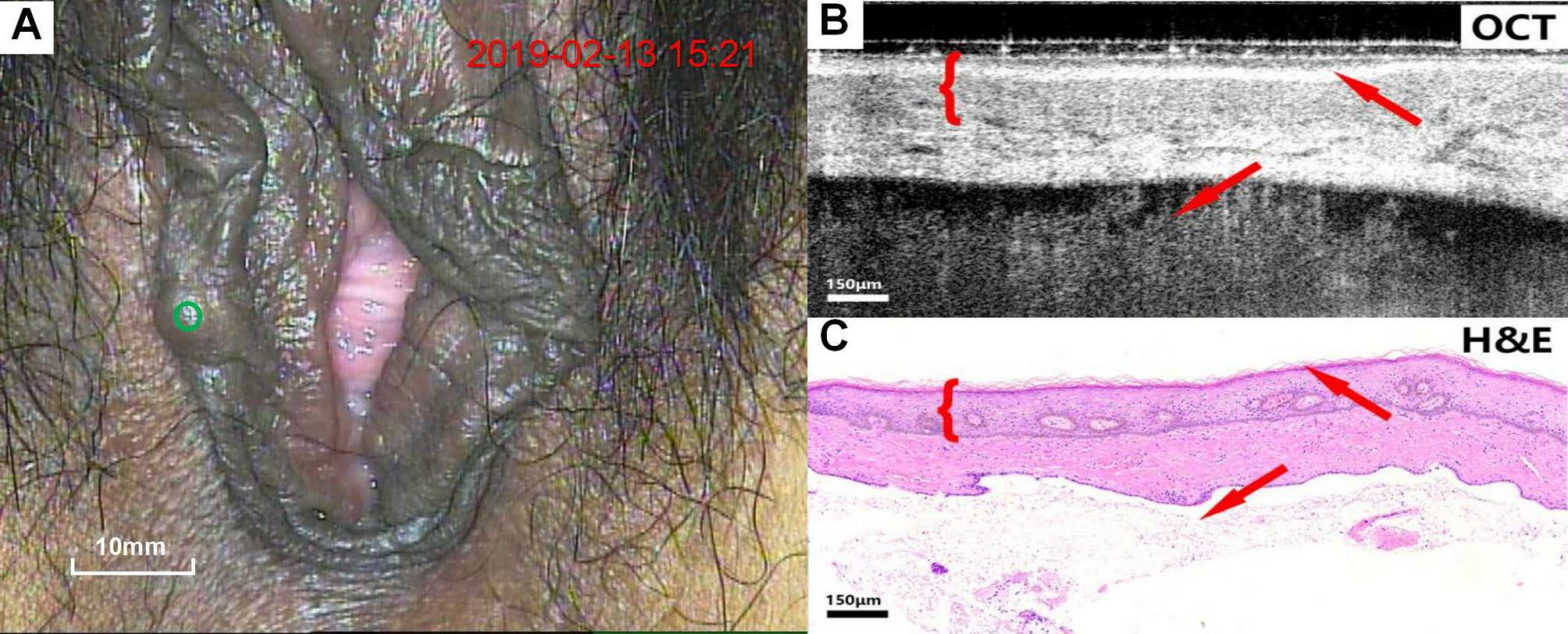
Colposcopy images, OCT images and H&E staining images of cyst. Scale bars 150 μm. In OCT image of a fibrous epithelial polyp of the vulval (Fig. 8C), numerous dark strip areas in the stroma were suspected to be capillaries produced by the lesion. The dark areas with clear boundaries could be clearly observed in the OCT image of the cyst (**C**), effectively corresponding to the cyst in the pathological tissue image (**C**).

Figures 4, 5, 6, 7, 8, 9 show colposcopy images, OCT images and the related H&E biopsies of the affected vulva, including condyloma acuminatum, papilloma, sclerosing lichen, atrophic sclerosing lichen, fibrous epithelial polyps, and cysts.

Figure 4A–C present colposcopy images, OCT images and H&E staining images of condyloma acuminatum. Condyloma acuminatum (Fig. 4C) had the pathologic appearance of a papillary projection of the epithelium (arrow) with hollowed out cells. The OCT image (Fig. 4B) presents the prominent papillary projections of the epithelium (arrow) with the overlying layer excessively keratinized, whereas the koilocyte is not easily identified.

Figure 5A–C presents colposcopy images, OCT images and H&E staining images of papilloma. Unlike condyloma acuminatum, papilloma H&E tissue sections (Fig. 5C) do not have typical hollowed-out cells, and the OCT images (Fig. 5B) also clearly show the typical papillary projections (arrows).

Figure 6A–C show colposcopy images, OCT images of lichen sclerosus and the corresponding H&E histological sections. In OCT images (Fig. 6B), the epithelium and stroma were well stratified, the overall scattering was uniform, the epithelial Angle was missing, and no serrated structure was identified. The surface layer highlighted is the keratinized layer (arrow).

Figure 7A–C show colposcopy images, OCT images of atrophic sclerosing lichen and the corresponding H&E histological sections. The OCT images of atrophic sclerosing lichen (Fig. 7B) also show well-stratified epithelium and stroma, disappearance of rete pegs, and flattening of the base. However, compared with normal epithelium, the thickness of the epithelium becomes significantly thinner as impacted by atrophy. Surface keratinization (arrow) could also be observed in the OCT image.

Figure 8A–C show colposcopy images, OCT images of fibroepithelial polyps and the related H&E tissue sections. Considerable capillaries are produced in the stroma. The OCT image (Fig. 8B) shows a significant increase in stripe dark areas (arrows) in the stroma, which were suspected to be capillaries. Passivation and flattening of the rete pegs further confirmed the pathological tissue.

Figure 9A–C show colposcopy images, OCT images of cysts and the related H&E tissue sections. The cyst displays significant features. The cyst in the stroma appeared in the OCT image (Fig. 9B) as a well-defined, low-scattering dark area (arrow below) that was easily identified. The epithelium (shown in braces) was clearly stratified from the stroma with uniform scattering. Due to the compression of cysts, the rete pegs disappear and flatten out. The surface keratinization (shown by the upper arrow) appeared as a bright area of high scattering.

### Data analysis

In this multicenter study, 63 patients from three hospitals were recruited in the statistical analysis. According to the overall diagnostic results, compared with the pathological results, the sensitivity, specificity and accuracy of the OCT examination reached 83.82%(95%CI72.5%-91.3%), 57.89%(95%CI34.0%-78.9%) and 78.16%, respectively. The sensitivity, specificity and accuracy of the colposcopy examination were 91.43%, 53.57% and 74.69%, which demonstrated that the OCT has high sensitivity and a great accuracy. 95% confidence intervals for proportions are calculated by using the efficient-score method (corrected for continuity) described by Robert Newcombe, based on the procedure outlined by E. B. Wilson in 1927. Moreover, due to the high resolution of the OCT system, the internal microstructure of the vulva could be clearly displayed, which matched well with the corresponding histological sections. Thus, by exploiting the mentioned feature of the OCT, vulvar lesions could be accurately identified and diagnosed. Table 3 summarizes the results of OCT diagnosis in the three hospitals.

**Table 3:** OCT diagnosis results.

Hospital	No. of patients	Sensitivity (95% CI)	Specificity (95% CI)	Accuracy (%)
The Third Affiliated Hospital of Zhengzhou University	20	82.35% (55.8%–95.3%)	75.0% (21.9%–98.7%)	80.95%
The First Affiliated Hospital of Xinxiang Medical College	24	82.14% (62.4%–93.2%)	66.7% (24.1%–94.0%)	79.41%
Luohe Central Hospital	19	86.95% (65.3%–96.6%)	44.4% (15.3%–77.3%)	75.00%
Overall	63	83.82% (72.5%–91.3%)	57.9% (34.0%–78.9%)	78.16%

## Discussion

In our multicenter study, 63 patients from three Chinese hospitals participated in the study, and the overall diagnostic results were as follows: Compared with pathological results, the sensitivity, specificity and accuracy of OCT were 83.82%, 57.89% and 78.16%, with the highest sensitivity of 86.95%(95%CI 65.3%-96.6%) obtained in Luohe Central Hospital. The highest specificity and accuracy were obtained in the Third Affiliated Hospital of Zhengzhou University, which were 75.0%(95%CI 21.9%-98.7%) and 80.95%, respectively. The specificity (44.4%) and accuracy (75.0%) of the Luohe Central Hospital were the lowest, and the sensitivity (82.14%) of the First Affiliated Hospital of Xinxiang Medical University were the lowest. In the final study data, the overall specificity was low, which may be related to the small sample size, and the difference in the sample size of the three hospitals may lead to the difference in OCT results. In addition, the specificity of the Third Affiliated Hospital of Zhengzhou University and Luohe Central Hospital had a large range of confidence intervals. In this study, the possible factors were analyzed, the OCT images incorrectly diagnosed by three researchers were rechecked, and some OCT image features were found to be not completely consistent with histopathological images. Careful analysis may be dependent of the following reasons :(1) the scan site of OCT may not be exactly the same as the biopsy site, since there is no such effective way to mark the exact scan site of OCT; (2) the quality of some OCT images deteriorates due to interference factors (e.g., bubbles in disposable latex covers), which may affect OCT classification results; (3) Impacted the limited experience of the three image researchers, some missed diagnosis and misdiagnosis may be caused. In “Conclusion”, the overall results showed that the OCT examination results were in good agreement with the pathological results.

The colposcopy was co-examined with the OCT in 273 of the 63 patients. Compared with the results of the colposcopy, the positive and negative results of the OCT were 89.9%, 60.7% and 75.5%, respectively. The Kappa value was 0.51, generally considered statistically to be highly consistent between 0.40 and 0.75. For this reason, the results of the two methods show a high consistency.

In Ronni Wessels et al.^22^'s study on the accuracy of appropriate surgical margin for the OCT diagnosis of surgical patients with vulval squamous cell carcinoma, the results are presented below. At the defined threshold, the sensitivity and specificity of the OCT were 100% and 80% when layer thickness was considered, and 100% and specificity were 70% when attenuation coefficient was considered. This suggested that OCT can be suitable for the diagnosis of vulvar diseases.

Differences were reported between this OCT study and pathological tissue section features, i.e., the hollowed-out cells of condyloma acuminatum can rarely be observed in the OCT images, which are similar to the optical characteristics of papilloma tissue and difficult to distinguish, whereas this does not affect the results of the two categories of classification. In addition, the OCT results may be inconsistent with the corresponding pathological findings. The reasons are presented as follows. (1) The scanning location of the OCT may not be identical to the location of biopsy sampling, and this difference leads to different outcomes. (2) The effect of mucus, bubble and other interference factors on the quality of some OCT images, and ultimately affect the results of the OCT.

The existing diagnosis of vulva diseases is mostly determined by clinical manifestations and physical examination (e.g., vulva pruritus or pain, vulva ulcers, vulva vegetations, rough skin and mucous membrane of the vulva, nodules and masses of the vulva, as well as vulva color changes). However, limitations exist in the gross diagnosis. In addition, some vulvar diseases have the risk of malignant transformation (e.g., vulvar sclerosing lichen)^23^. Accordingly, vulva diseases should be identified and treated early. However, simple colposcopy examinations lack established usefulness for vulvar diseases^24^. Though the colposcopy combined with biopsy can exhibit a high diagnostic accuracy^25^, it is an invasive examination that is difficult to serve as a screening method for clinical vulvar diseases.

The OCT, a non-invasive, high-resolution, real-time and rapid imaging technology, has become a research hotspot. However, the OCT technology fails to clearly image deeper tissues, and it has high professional requirements to judge collected images. The existing research on vulvar diseases remains at the early stage. However, as the OCT equipment is leaping forward, and the technical level is being elevated, the OCT will be of important clinical significance for screening and diagnosing vulvar diseases, and it will exhibit immeasurable advantages and wide application prospects in medical detection and disease diagnosis.

## Conclusion

In this multicenter clinical study, vulvar diseases were assessed by the non-invasive and real-time examination with a high-resolution OCT system. As indicated by the results, the OCT could produce relatively accurate diagnostic results, while providing high resolution microscopic images consistent with histological features. This study verified the validity of the OCT technique in the diagnosis of vulvar lesions. The OCT is expected to be a novel screening method for vulvar lesions.
